# Editorial: Intergenerational Health Inequalities

**DOI:** 10.3389/fpubh.2022.888995

**Published:** 2022-04-13

**Authors:** Heather Brown, Peter Eibich, Olga Biosca

**Affiliations:** ^1^National Institute for Health and Welfare, Helsinki, Finland; ^2^Population Health Sciences Institute, Newcastle University, Newcastle upon Tyne, United Kingdom; ^3^Labor Demography, Max Planck Institute for Demographic Research, Rostock, Germany; ^4^Glasgow Caledonian University, Glasgow, United Kingdom

**Keywords:** intergenerational effects, editorial, public health, health inequalities, families

Intergenerational inequalities in health are an avoidable cause of lifetime poor health. In order to develop policy and interventions to ensure equity of opportunity it is important to understand the causes and effects of intergenerational health inequalities and how they can be reduced. This Research Topic gathers a range of international contributions on intergenerational health relationships and their implications for health inequalities. This is a multidisciplinary collection using both qualitative and quantitative techniques to understand intergenerational health relationships and how interventions can be developed to improve health across different generations and reduce health inequalities. The need to consider the costs and benefits not only to current but to future generations when developing public health policy has become apparent from the current crises facing humanity, such as climate change and the Covid-19 pandemic. In this last case, the burden and cost of the measures designed to contain the virus (e.g., school closures) has differential impacts and long-term consequences across the generations. Thus, this collection offers a starting point to understand different perspectives for evaluating the relationships between generations and what this means for the development of policy and health inequalities.

The first paper by Wickham and Fanecourt develops an ethical framework to explore how reforms to welfare policy and, in particular, cuts to social security spending may have intergenerational effects. Policy makers developing social security policy need to consider the health effects of reducing benefits. These changes may impact on the health and safety not only of individual recipients but of their children. Thus, there may be long term consequences on health and inequalities of contractionary welfare policy.

The second paper by Vera-Toscano and Brown uses data from the Household Income and Labor Dynamics of Australia Survey from 2001 to 2019 to explore the intergenerational correlation in physical and mental health between young adults (aged 25–35) and their parents. The analysis explores how childhood disadvantage, childhood health, educational attainment and social mobility relate to the correlation in health between parents and their young adult children. Childhood disadvantage is the only factor that influences the correlation in parental and offspring health. These results relate back to the first article by Wickham and Fanecourt by demonstrating that poverty has long term consequences for health and inequalities across generations.

The next article by Crone et al. employed an intergenerational perspective to evaluate a family engagement approach for children with obesity. The authors utilized a mixed method approach of interviews, questionnaires, and an *n*-of-1 study on 12 children and their parents over a 12-week period in the Netherlands. The results suggest that for those families that responded there was some improvement in child's physical activity and mothers reported being more energetic. These findings highlight the potential importance of involving the whole family to support a healthy weight.

The final article by Xu and Luo takes a different angle to Vera-Toscano and Brown and investigates the association between adult children's educational attainment and their elderly parents' cognitive health in China using data from the China Health and Retirement Longitudinal Survey. They explore the role of intergenerational support in explaining this relationship. The authors find a significant association between children's educational attainment and their elderly parents' cognitive health but the mechanisms vary by the adult child's gender. These findings highlight how investment in children can have positive influences on the health of parents in old age.

Taken together, these studies highlight how health is shaped within the context of the family, and how social policies affecting one generation can have long-lasting consequences for both younger and older family members. We hope that the reader will find this a useful collection to think about the connectiveness of health across generations, and that it might inspire future research on interventions that might acknowledge and support these connections to improve health and reduce health inequalities.

## Author Contributions

HB drafted the editorial. All authors commented on the manuscript and agreed the final submission.

## Conflict of Interest

The authors declare that the research was conducted in the absence of any commercial or financial relationships that could be construed as a potential conflict of interest.

## Publisher's Note

All claims expressed in this article are solely those of the authors and do not necessarily represent those of their affiliated organizations, or those of the publisher, the editors and the reviewers. Any product that may be evaluated in this article, or claim that may be made by its manufacturer, is not guaranteed or endorsed by the publisher.

